# Long-term tooth survival and success following primary root canal treatment: a 5- to 37-year retrospective observation

**DOI:** 10.1007/s00784-023-04938-y

**Published:** 2023-03-18

**Authors:** Isabel López-Valverde, Fabio Vignoletti, Gianfranco Vignoletti, Conchita Martin, Mariano Sanz

**Affiliations:** 1 Section of Periodontology, Faculty of Odontology, University Complutense of Madrid, Madrid, Spain; 2 Studio Dentistico Vignoletti, Private practice, Verona, Italy

**Keywords:** Primary root canal treatment, Endodontic treatment, Tooth survival, Endodontic success, Long term

## Abstract

**Objectives:**

The aims of the present longitudinal retrospective observational case series study were to investigate the survival and success rates of primary non-surgical endodontic therapy.

**Materials and methods:**

Patients with at least one endodontically treated tooth (ETT), with 5 years of follow-up and in compliance with the recall programme of at least 1 time per year in a private practice setting, were recruited. Kaplan-Meier survival analyses were performed considering (a) tooth extraction/survival and (b) endodontic success as the outcome variables. A regression analysis was performed to evaluate prognostic factors associated with tooth survival.

**Results:**

Three hundred twelve patients and 598 teeth were included. The cumulative survival rates showed 97%, 81%, 76% and 68% after 10, 20, 30 and 37 years, respectively. The corresponding values for endodontic success were 93%, 85%, 81% and 81%, respectively.

**Conclusions:**

The study demonstrated high longevity in symptomless function as well as high success rates of ETT. The most significant prognostic factors associated with tooth extraction were the presence of deep (> 6 mm) periodontal pockets, the presence of pre-operative apical radiolucency and the lack of occlusal protection (no use of a night guard).

**Clinical relevance:**

The favourable long-term (> 30 years) prognosis of ETT must encourage clinicians to rely on primary root canal treatment when taking the decision regarding whether a tooth with pulpal and/or periapical diseases should be saved or be extracted and replaced with an implant.

## Introduction

Endodontic treatment is a highly predictable therapy for preserving the natural dentition, with demonstrated high long-term survival and success rates [1]. However, the lack of consistency in defining success and failure after endodontic therapy has resulted in reported results with considerable heterogeneity [2]. When endodontic success has been based on either strict or loose criteria, the pooled success rates have ranged between 74.7% (95% CI: 69.8–79.5%), when using strict radiographic and clinical criteria, and 85.2% (95% CI: 82.2–88.3%), when using loose criteria. Similarly, analyses by meta-regression showed that the reported success rates were 10.5% lower when based on strict criteria, compared to the application of loose success criteria [3]. These differences in the appraisal of success or failure hampered an effective assessment of the efficacy of endodontic therapy, and due to this, current trends in endodontic research focus on reporting tooth survival as well as success rates. Tooth survival after root canal treatment (RCT) has been defined as the continued presence of the tooth in function, provided it is symptom-less [4]. Using this definition, a systematic review based on randomised clinical trials reported that the pooled probability of long-term (4–5 and 8–10 years) tooth survival after RCT ranged between 93 and 87%, respectively [1].

The prognostic factors associated with survival and success rates after RCT have also been of interest to both researchers and clinicians and have been broadly categorised, according to the time frame in relation to the RCT, as (I) pre-operative, including (a) patient-related factors, such as age, gender, medical status or ethnic origin; (b) operator-related factors, such as skill and experience; and (c) tooth-dependent factors, such as the health condition of the tooth, pulp or periodontium; (II) intra-operative, mainly related to the quality of the RCT, such as inadequate obturation, short root canal fill or overfill; and (III) post-operative, mainly related to restorative aspects.

Preoperatively, the absence of a periapical radiolucency; intra-operatively, the presence of root filling without voids and extending up to 2 mm within the radiographic apex; and post-operatively, the quality of the coronal restoration were the most significant prognostic factors for endodontic success identified in a systematic review [5]. In terms of survival, the lack of a crown restoration after RCT, whether the treated tooth had mesial and distal proximal contacts, whether the tooth was functioning as an abutment for removable or fixed prosthesis and whether it was a molar, have been identified as the most significant factors for failure after RCT [1]. Furthermore, long-term observations have shown that tooth loss due to non-endodontically related causes, such as periodontitis, caries and vertical root fractures, may significantly influence the survival of endodontically treated teeth (ETT) [6–9].

Studies evaluating the long-term (> 20 years) survival and success of ETT are very few with no data exceeding 30 years of follow-up. It is, therefore, the primary objective of this retrospective observational study to evaluate the long-term survival rates of ETT. The secondary objectives aimed at evaluating (i) prognostic factors associated with tooth survival and (ii) long-term endodontic success .

## Materials and methods

### Study design

This is a longitudinal retrospective observational case series study based on a sample of patients with ETT treated by an experienced endodontist (GV) in a private dental practice in Verona, Italy, between 1979 and 2016.

Because of the use of only retrospective fully anonymized data, this study was a nonintervention clinical trial, adopting a standardized nonexperimental protocol, without the need for local review board approval according to the European Guidelines for Good Clinical Practice (CPMP/ICH/135/95).

The manuscript was written in accordance with the recommendations for reporting clinical case series studies [10].

**Fig. 1:**
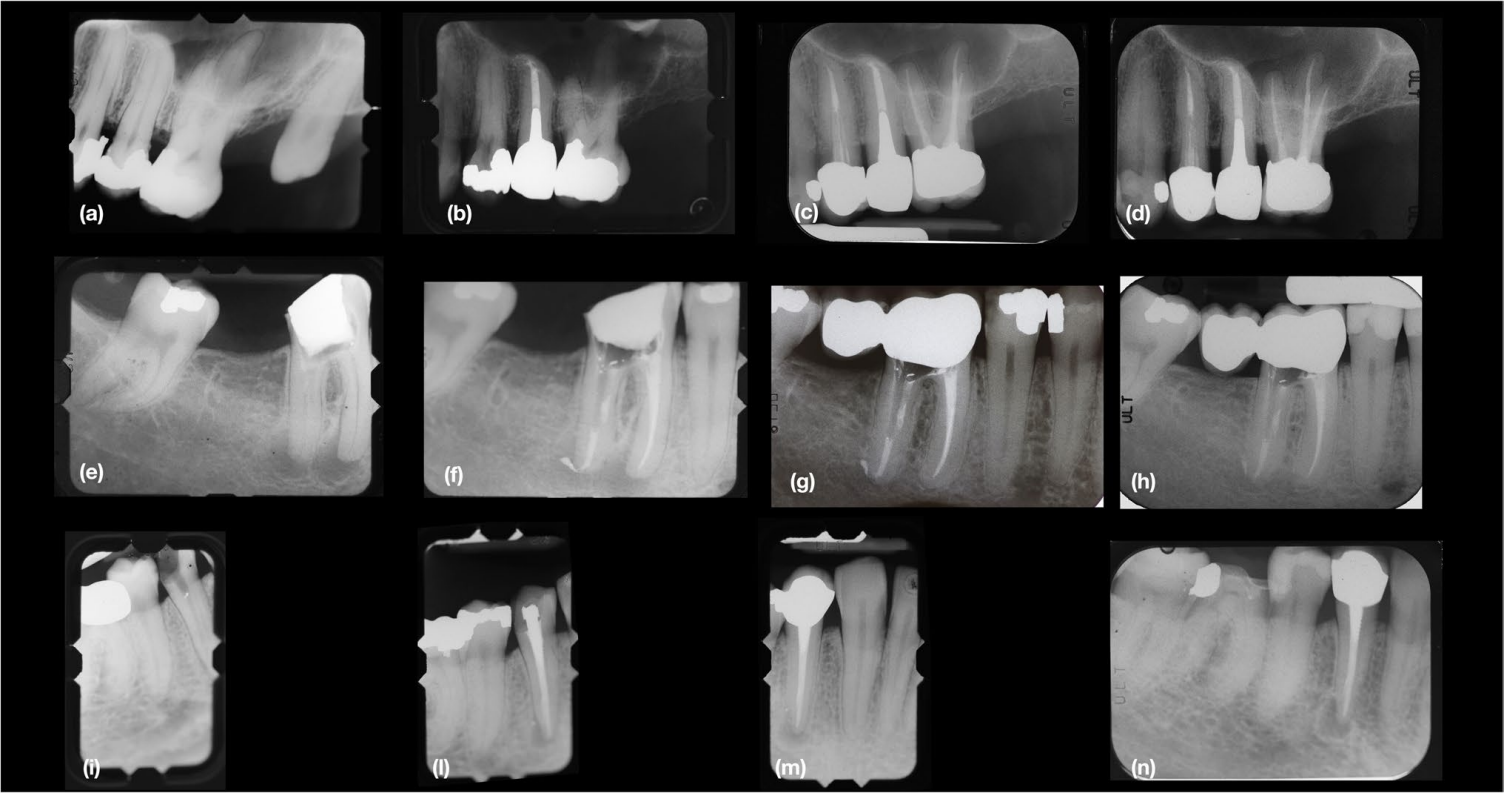
(**a**), Tooth 2.5. pre-op periapical radiograph, year 1979. (**b**), Periapical radiograph after endodontic and restorative treatment with cast metal post and crown, year 1979. (**c**), Post-op periapical radiograph follow-up, year 2004. (**d**), Post-op periapical radiograph demonstrating success 37 years after primary endodontic treatment , year 2016. (**e**) Tooth 4.6. pre-op periapical radiograph, year 1982. (**f**), Periapical radiograph after endodontic treatment, year 1982. (**g**), Tooth 4.6. post-op periapical radiograph with restorative treatment with a fiber post and radiolucent composite material and a crown, follow-up year 2000. (**h**), Tooth 4.6. post-op periapical radiograph demonstrating success after 34 years follow-up, year 2016. (**i**), Tooth 4.4. pre-op periapical radiograph, year 1979. (**l**), Periapical radiograph after endodontic treatment, year 1979. (**m**), Post-op periapical radiograph follow-up with restorative treatment, year 2004. (**n**), Post-op periapical radiograph demonstrating survival in painless function 37 years after primary endodontic treatment, year 2016.
- *Recall programme*
  After the RCT and tooth restoration, patients entered a recall programme at intervals that varied between 1 to 4 times per year, depending on their periodontal status and the degree of the patient’s oral hygiene. At each periodic recall visit, a dentist or dental hygienist provided professional supra-gingival biofilm removal, as well as reinforced oral hygiene instructions. If indicated, sub-gingival instrumentation with ultrasounds and curettes was carried out. Approximately every 2 years, based on the ‘as low as reasonably achievable optimisation’ concept, peri-apical radiographs were taken in the posterior quadrants to monitor the occurrence of caries and periodontitis. Selective grinding and control of the occlusal night guard were also performed when needed.

These patients received the following pre-operative, intra-operative and post-operative treatment protocols:

- *Clinical and radiographic evaluations*
  Patients were clinically and radiographically examined by a single endodontist (GV) who established the treatment plan. The periodontal evaluation included the presence of plaque and bleeding on probing, probing pocket depths (PPD), marginal recession and clinical attachment levels (CAL), registered in 6 sites per tooth with a manual periodontal probe (PCP-UNC 15, Hu-friedy). Furthermore, bruxism was diagnosed clinically and from study casts by evaluating the presence of tooth wear. Full-mouth periapical radiographs were taken with the paralleling technique using a long cone with a ring XCP holder.
- *Initial preparation and treatment planning*
  Patients received periodontal therapy including oral hygiene instructions, supra and subgingival root instrumentation and periodontal surgery, depending on the periodontal diagnosis. If indicated by the degree of bruxism, an occlusal night guard was applied.
- *Endodontic and restorative treatment*
  Between 1979 and 1995, teeth requiring RCT had their root canals prepared and filled using the Schilder technique [11–13]. In brief, after isolation with a rubber dam, the pulp chamber was opened with a diamond bur (Intensiv 206, Intensiv, Grancia, Switzerland), and the canals were identified. These were scouted by using #08, #10 and #15 stainless steel files before preflaring with #1 and #2 Gates burs. The working length was determined on periapical radiographs at 0.5 mm coronal to the radiographic apex until 1991 and, thereafter, using an electronic apex locator (Osada Electric Co., LTD. Apit or Endex, Japan) at ‘0’ reading position. The ‘step-back’ procedure was then adopted using a sequence of stainless-steel files. Gutta-percha was compacted vertically with heat carriers (OP and OOP; from 1991 Touch’n Heat, Analytic Technologies, Redmond, WA, USA and from 2000 with System B, Sybronendo). Back packing was accomplished with the Obtura syringe (Obtura Spartan, Foothill Ranch, CA, USA).
  From 1995 onwards, canals were prepared using a rotatory technique. The coronal third of the canal was enlarged with Gates Glidden drills 1 and 2 followed by a modified batt drill (Dentsply Maillefer, Ballaigues, Switzerland), while the apical portion was instrumented with pre-curved stainless-steel Hedström files (Dentsply Maillefer) with increasing diameters up to 20, without apical pressure. The apical diameter was recorded using light-speed instruments (Lightspeed LSX Instrument, Discus dental, Culver City, CA, USA). The largest diameter that could not bypass the working length was chosen as the apical diameter. Then, a step-back technique with increasing diameters of light-speed instruments was used to reach the previously prepared coronal third. All canals, except those presenting exudation, were closed in one session, which were provisionally medicated with calcium hydroxide. Gutta-percha cones were used to obturate the canals. The master cone was dipped into cement (Pulp Canal Sealer™, Kerr until 2000 and after that AH Plus™, Dentsply) and well adapted to the canal. Accessory gutta-percha cones where introduced. Excess gutta-percha was removed and vertically compacted with a heat carrier (Touch’n Heat or System b) and endodontic compactors (Thompson Dental Meg CO USA Tactile Tone SS Dr. Vignoletti plugger). In both techniques, 3.5% sodium hypochlorite was used as a disinfectant.
  Upon completion of RCT, a direct filling or a permanent core was placed in the access cavity, depending on the remaining tooth structure and the operator’s choice. Similarly, the choice of restorative therapy depended on the remaining tooth structure. If cusp coverage was needed, a crown or onlay was constructed. If a cast metal (precious alloy) post and core or a fibre post were required, the gutta-percha root filling was cut back leaving at least 4 mm of root filling apically. Then, the tooth was restored with a temporary post-retained crown, and once manufactured, the definitive restoration was cemented.
  A final radiograph was taken after the restoration, and then, subsequent control radiographs were taken at 1 year and, thereafter, every 2 years (Fig. 1).

### Selection of patients and ETT (Fig. 2)

Patients and ETT were eligible when fulfilling the following inclusion criteria:

- Adults (> 18 years) with at least one endodontically treated tooth after at least 5 years of follow-up.
- Patients in compliance with the recall programme within at least 1 time per year.
- Teeth with baseline pre-operative and post-operative clinical and radiographical data.

**Fig. 2:**
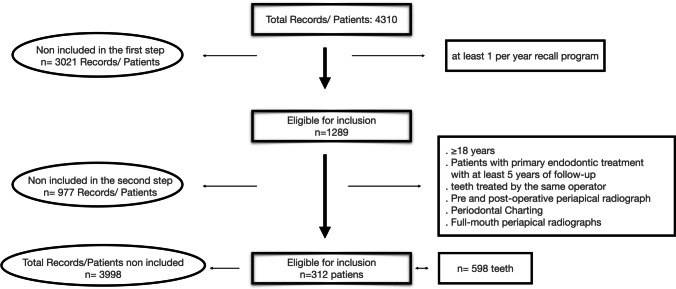
Flow chart of patient inclusion in the study

Teeth were excluded if:

- Treated with endodontic surgery, endodontic re-treatment, apexogenesis or apexification.
- Third molars.

### Data collection

One researcher (ILV) screened 4310 dental records and selected patients treated up to the year 2016 using these referred criteria. The relevant pre-operative, intra-operative and post-operative data were transferred into an Excel data sheet. Potential prognostic factors such as age, sex, diagnosis of bruxism, tooth type and position, probing pocket depth (PPD), pulp status (either necrotic or vital), presence of radiographic radiolucency, type of restoration and posts were registered (Table 1). In detail, pulp status was diagnosed with an electric pulp tester (Analytic Technology). Necrotic status was recorded when the electric pulp tester value was 80. Pre-operative radiographic radiolucency was scored as positive whenever any sign of apical or lateral radiolucency was evidenced on baseline radiographs. Probing pocket depth (PPD) represented the deepest recorded periodontal probing of the tooth after RCT.

**Table 1:** Independent variables of the entire sample of 598 teeth, expressed as the absolute value (*n*) or proportion (%). At the patient level, out of 312 patients, 45.5% of the patients were men, and 54.5% were women. Out of these, 119 (38.1%) were diagnosed with bruxism, and 69 (22.1%) used a night guard. The mean age of the sample was 65.5 years (SD: 14.07). * *n* (87) refers to the number of teeth extracted for each category, ** *n* (73) refers to the number of unsuccessful teeth in each category

Independent variables	*n* (598)	%	*n (87) Extraction	**n (73) No success
Gender				
Female	324	54.2	45	29
Male	274	45.8	42	44
Bruxism				
Yes	215	36	25	27
No	383	64	62	46
Night guard				
Yes	123	20.6	8	12
No	475	79.4	79	61
Dental arch				
Maxillary	347	58	51	40
Mandibular	251	42	36	33
Type of tooth				
Incisors	112	18.7	15	6
Maxillary	78	13	9	2
Mandibular	34	5.7	6	4
Canines	44	7.4	11	5
Maxillary	28	4.7	7	3
Mandibular	16	2.7	4	2
Premolars	175	29.3	22	24
Maxillary	92	15.4	14	12
Mandibular	83	13.9	8	12
Molars	267	44.6	39	38
Maxillary	149	24.9	21	29
Mandibular	118	19.7	18	9
Endodontic treatment				
Manual	199	33.3	46	33
Rotatory	399	66.7	39	40
Pulp status				
Vital	429	71.7	59	45
Necrotic	169	28.3	28	28
Pre-operative radiographic radiolucency				
Yes	295	49.3	57	53
No	303	50.7	30	20
Post-operative radiographic radiolucency				
Yes	22	96.3	82	22
No	576	3.7	4	51
Probing pocket depth				
(1–3 mm)	238	39.8	36	7
(4–5 mm)	263	44	33	21
(≥ 6 mm)	97	16.3	18	45
Fibre post				
Yes	193	32.3	15	17
No	405	67.7	72	56
Cast metal post (precious alloy)				
Yes	93	15.6	30	16
No	505	84.4	57	57
Restoration				
Direct filling	182	30.4	28	11
Single crown	217	36.2	28	31
Dental bridge	197	32.9	29	29
Overdenture	2	0.3	2	2
Adjacent tooth				
Not present	234	39.1	48	38
Natural tooth	220	36.8	21	21
Crown or bridge	125	20.9	17	14
Overdenture or removable partial denture	0	0	0	0
Implant supported crown	19	3.2	1	0
Overlay/onlay	0	0	0	0
Pontic	0	0	0	0
Antagonist:				
Not present	22	3.7	4	4
Natural tooth	337	56.4	35	32
Crown or bridge	193	32.3	39	37
Overdenture or removable partial denture	4	0.7	3	0
Implant supported crown	13	2.2	0	0
Overlay/onlay	4	0.7	1	0
Pontic	25	4.2	5	0

### Outcome variables

*Tooth survival* (primary outcome) was based on maintenance of the ETT, provided it was painless and in function [14]. At the patient level, whenever a tooth was extracted, the patient entered the category ‘no survival’.

*Tooth mortality* was accounted when the ETT was extracted. Extraction time was recorded as the time interval (measured in years) between the end of the RCT and the extraction date. The reason for tooth extractions were recorded and were divided into the following categories: (I) untreatable caries, (II) fracture of the crown, (III) vertical root fracture, (IV) periodontal disease progression and (V) endodontic inflammation. In detail, teeth were extracted when the decay or fracture of the crown was incompatible with the tooth restoration, when a vertical root fracture was diagnosed and the tooth was incompatible with function and comfort and when the residual periodontal support was incompatible with function and comfort and could not be improved with additional periodontal therapy.

*Endodontic success* (secondary outcome) was evaluated clinically and radiographically using the criteria defined in the Toronto study [14]. In brief, success or ‘healed’ was defined as absence of any radiographic sign of apical periodontitis with a concomitant lack of any clinical signs and symptoms.

### Statistical analysis

Data *a*nalysis was performed using both the subject (survival analysis) and the tooth as the statistical unit. Descriptive statistics utilized means and standard deviations (SD) and 95% confidence intervals (CI), as well as proportions, depending on the type of variable (quantitative or qualitative). A Kaplan-Meier survival analysis was performed considering (a) tooth extraction/survival and (b) endodontic success as the outcome variables, and different tooth-related factors were assessed: tooth type (incisor, canine, premolar and molar), location (mandible vs maxilla) and position.

A regression analysis was performed to evaluate risk indicators associated with tooth extraction/survival. To identify the variables to include in the logistic regression model, a bivariate chi-square analysis was performed with all the independent variables (Table 1), and if significant in this first step, they were included in the final regression analysis. Logistic regression results were presented as odds ratios (OR). Survival curves were further constructed to compare survival distributions according to the identified risk indicators. The long-rank test (Mantel-Cox test) was used to compare these survival distributions, and the *p* value was adjusted for multiple comparisons.

IBM SPSS version 22 (SPSS Inc., Chicago, IL) was used for statistical analyses, and the level of significance was set at *p* ≤ 0.05.

## Results

### Study population

The characteristics of the study sample are presented in Table 1. Three hundred and twelve patients with a mean age of 65.5 (SD 14.07) and 598 teeth were included in the study. Between 1979 and 1994, 199 (33.3%) teeth were treated according to the Schilder’s technique, whereas between 1995 and 2016, 399 (66.7%) were treated according to a crown-down rotary technique. The majority of ETT were molars (267; 44.7%), followed by premolars (175; 29.3%), incisors (112; 18.7%) and canines (44; 7.4%). Regarding the type of restoration, the vast majority (414; 69.1%) were restored with prosthetic crowns, whereas 182 (30.4%) were direct amalgam or composite fillings.

### Tooth survival

**Fig. 3:**
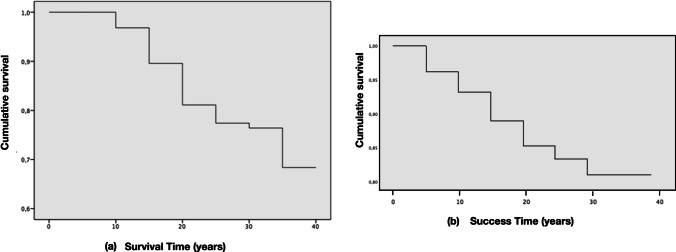
Kaplan–Meier function curves of 598 endodontically treated teeth. (**a**) Survival and (**b**) Endodontic Success

The overall survival rate after a mean follow-up period of 21 years was 85.5% (511 out of 598; 95% CI: 81% to 88%) and 81.7% (255 out of 312; 95% CI: 77% to 85%) at the tooth and patient levels, respectively. The cumulative survival rate expressed by the Kaplan-Meier analysis curve showed that the probability of a tooth surviving 10, 20, 30 and 37 years after endodontic treatment was 97%, 81%, 76% and 68%, respectively (Table 2, Fig. 3a). The mean estimated tooth survival was 31.64 years (95% CI: 30.62 to 32.66) for both arches, 31.6 years (95% CI: 30.27 to 32.92) for the maxillary teeth and 31.76 years (95% CI: 30.21 to 33.32) for the mandibular teeth. Tooth survival was not affected by tooth position (*p* = 0.679), tooth type (molars, premolars, cuspids, incisors; *p* = 0.362) and whether the tooth was in the mandible or maxilla (*p* = 0.758).

**Table 2:** Mortality table for the survival of ETT. The number of terminal events equals the number of teeth lost in each interval

Interval start time (years)	Number of teeth entering in each time interval. Survival	Number of teeth withdrawn during this interval	Number of teeth exposed to risk	Number of terminal events. Survival	Probability of surviving (%)	Cumulative survival at the end of each interval	95% CI of cumulative success proportion	
							Lower limit	Upper limit
0	598	0	598	0	100	1.00	1	1
5	598	62	567	18	97	.97	0.9504	0.9896
10	518	180	428	32	93	.90	0.8804	0.9196
15	306	83	264	25	91	.81	0.7708	0.8492
20	198	90	153	7	95	.77	0.7308	0.8092
25	101	45	78	1	99	.76	0.7012	0.8188
30	55	34	38	4	89	.68	0.6016	0.7584
35	17	17	8	0	100	.68	0.6016	0.7584

### Tooth mortality

**Fig. 4:**
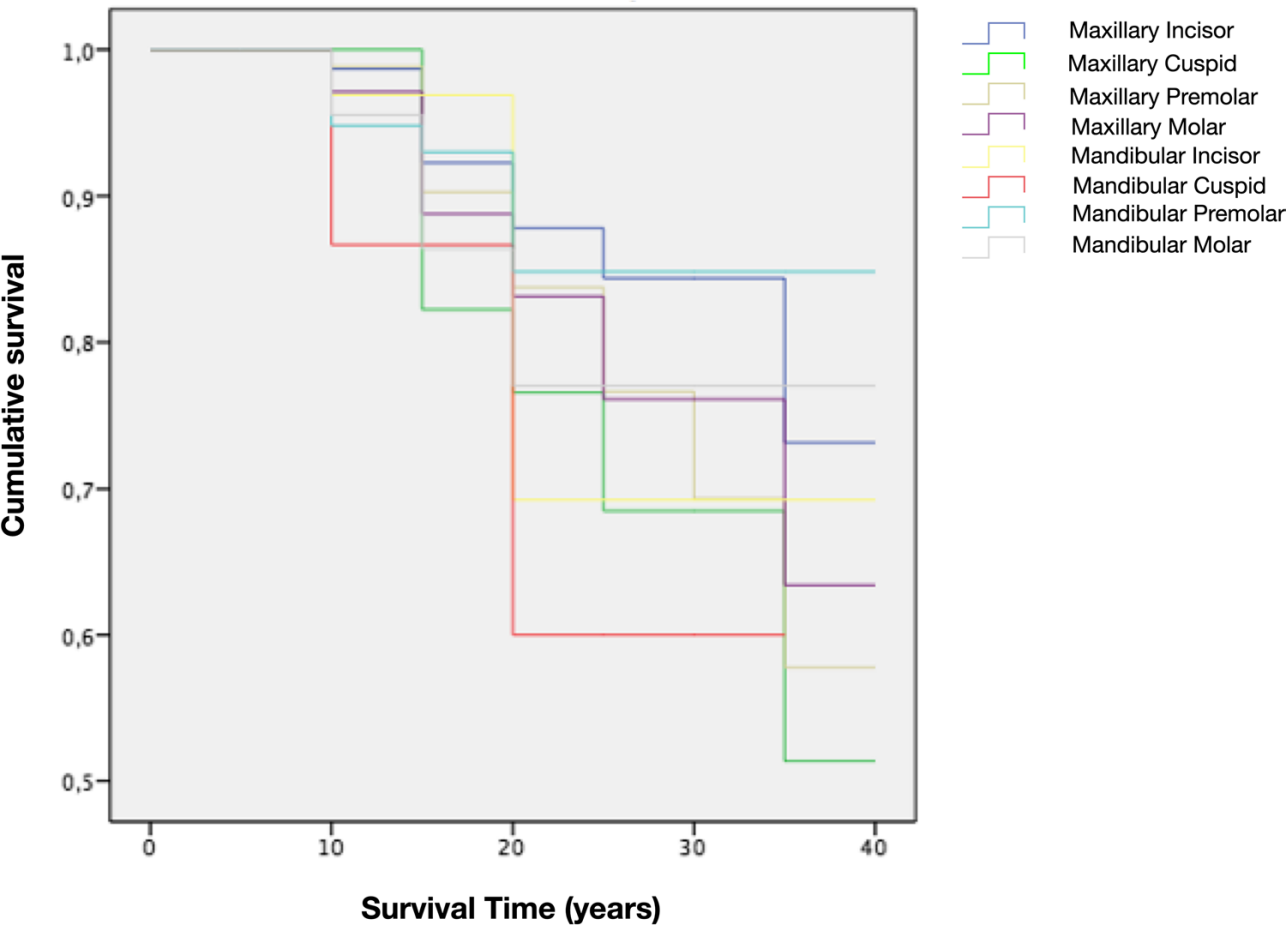
Kaplan–Meier function survival curves based on tooth position

Eighty-seven teeth representing 14.5% of the entire sample were extracted after an overall mean time of 14.1 (95% CI: 12.84 to 15.37) years. When analysing the Kaplan-Meier curves by tooth type, all teeth presented similar survival rates (approximately 80%) up to 20 years post-endodontic treatment. Afterwards, mortality rates varied considerably with maxillary cuspids being the most frequently lost teeth (53.7% survival rate), whereas mandibular premolars were the least (85% survival) (Fig. 4). The reasons for tooth extraction were caries (1.7%), fracture of the crown (2.2%), vertical root fracture (4.8%) and periodontal disease progression (5.9%) with no statistically significant differences (*p* = 0.671) (Table 3).

**Table 3:** Causes of extraction (absolute value, % of the extracted sample and total sample), mean (95% CI), and median time to extraction (IQR)

	*n*	% of the extracted sample	% total sample	Mean time to extraction (years)	95% CI (lower)	95% CI (upper)
Causes of extraction		100	14.6	14.103	12.842	15.365
Vertical root fracture	29	33.3	4.8	14.069	11.718	16.420
Periodontal disease progression	35	40.2	5.9	13.429	11.887	14.971
Caries	10	11.5	1.7	15.900	10.990	20.810
Crown fracture	13	15	2.2	14.615	10.839	18.392
Endodontic inflammation	0	0	0	0	0	0

#### Prognostic factors for extraction/survival

Table 4 depicts the chi-square analysis indicating the variables used for the logistic regression analysis. PPD ≤ 5 mm (OR = 0.68; 95% CI: 0.54–0.86; *p* = 0.001), use of a night guard (OR = 0.34; 95% CI: 0.13–0.86; *p* = 0.023) and use of a fibre post (OR = 0.47; 95% CI: 0.24–0.91; *p* = 0.026) were protective factors associated with tooth survival. Conversely, the presence of a cast metal post (OR = 2.14; 95% CI: 1.14–4.01; *p* = 0.018) and pre-operative presence of periapical radiolucency (OR = 1.87; 95% CI: 1.07–3.28; *p* = 0.028) were factors significantly associated with tooth extraction.

**Table 4:** The bivariate (chi-square) and logistic regression analyses for survival/extraction. Statistically significant variables according to the bivariate chi-square (*) analysis were included in the logistic regression analysis. Significant risk indicators are expressed as odds ratio (OR) (*). B: logistic regression coefficient (the exponentiation of the B coefficient, EXP(B), is the odds ratio). Comparisons of survival distributions according to the significant risk indicators were performed using the log-rank test (significant differences are expressed as **). The level of significance was set at *p* < 0.005.

	Chi-square	*p* value (chi-square)	B (logarithm of the OR)	*p* value (logistic regression)	OR EXP(B)	95% CI for OR (lower)	95% CI for OR (upper)	Log-rank test
Age	3.692	0.055						
Gender	0.247	0.619						
Dental arch	0.015	0.204						
Diagnosis of bruxism	2.303	0.129						
Use of night guard	8.061	0.005*	−1.088	0.023*	0.337	0.132	0.860	0.036**
Pulp status	0.773	0.379						
Radiographic radiolucency	9.210	0.002*	0.628	0.028*	1.874	1.071	3.277	< 0.001**
Radiographic healing	1.229	0.268						
Fiber_post	10.526	0.001*	−0.756	0.026*	0.469	0.242	0.912	0.425
Cast Metal_post	27.782	0.000*	0.759	0.018*	2.137	1.137	4.015	0.082
PPD < 5 mm	18.411	0.000*	-0.387	0.001*	0.679	0.538	0.858	< 0.001**
Single unit crown	1.805	0.179						
Dental bridge	18.307	0.000*	0.400	0.766	1.492	0.107	20.777	
Dental bridge with extension	1.720	0.190						
Dental bridge without extension	16.948	0.000*	0.239	0.855	1.271	0.097	16.726	
No adjacent tooth	11.000	0.001*	0.289	0.403	1.334	0.678	2.625	
Adjacent natural tooth	7.007	0.008*	0.079	0.846	1.082	0.488	2.402	
Adjacent crown or bridge	0.640	0.424						
No antagonist	3.348	0.067						
Antagonist natural tooth	10.762	0.001*	−0.428	0.128	1.082	0.488	2.402	
Antagonist crown or bridge	0.875	0.675						

**Fig. 5:**
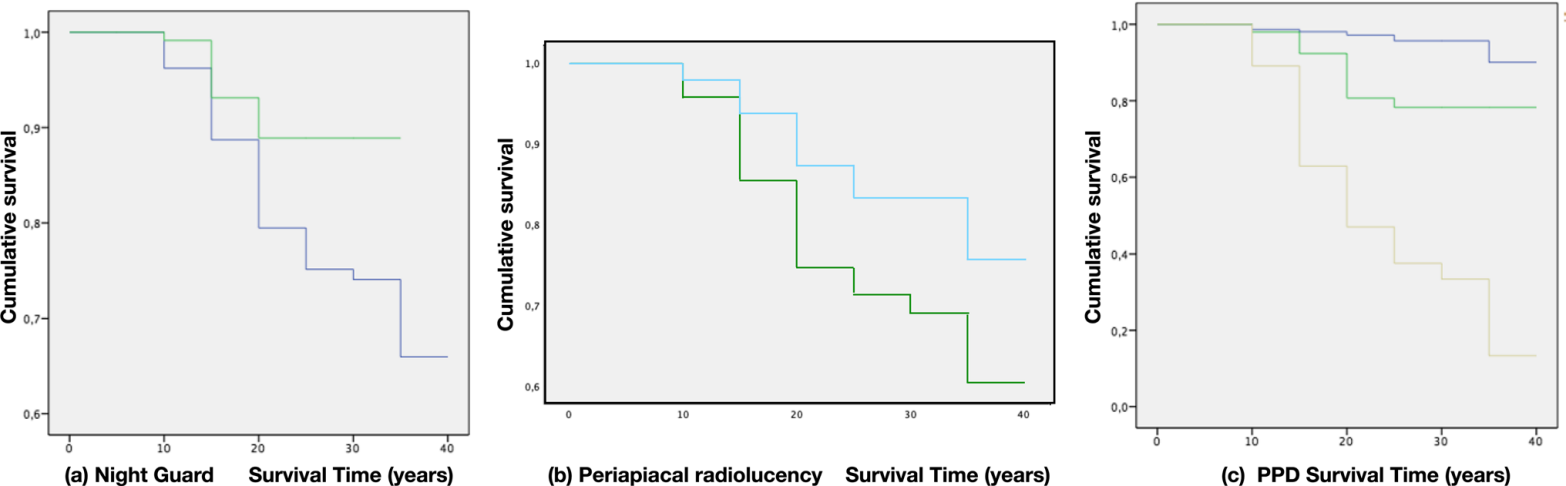
Kaplan–Meier function survival curves (**a**) comparing patients using (green line) or not using (blue line) the night guard, (**b**) comparing teeth with (green line) or without periapical radiolucency (blue line) and (**c**) comparing teeth with 1-3 mm PPD (blue line), 4-5 mm PPD (green line) and > 6 mm PPD (yellow line)

Survival curves were further constructed to evaluate survival distributions comparing the identified risk indicators. Only PPD < 5 mm, the presence of periapical radiolucency and use of a night guard demonstrated statistically significant differences (Table 4, Figs. 5a–c and 5).

#### Endodontic success

The overall success rates of ETT were 87.8% (95% CI: 84 to 90%) and 80.8% (95% CI: 75 to 86%) at the tooth and patient levels, respectively. Cumulative success expressed by the Kaplan-Meier curve analysis showed that treatment success rates at 10, 20, 30 and 37 years were 93%, 85%, 81% and 81%, respectively (Table 5, Fig. 3b). Success rates were not affected by tooth position (ranging from 97.4% for upper incisors to 84.6% for upper molars, *p* = 0.210), tooth type (molars: 85.8%, premolars: 86.3%, cuspids: 88.6%, incisors: 94.6%, *p* = 0.068) and whether the tooth was in the mandible (86.9%) or maxilla (88.5%) (*p* = 0.413). Only when comparing incisors versus molars, and maxillary incisors versus mandibular molars, was a tendency towards significance observed (*p* = 0.09 and *p* = 0.056, respectively).

**Table 5:** Mortality table for the success of ETT. The number of terminal events equals the number of teeth lost in each interval

Interval start time (years)	Number of teeth in each time interval. Success	Number of teeth withdrawn during this interval	Number of teeth exposed to risk. Success	Number of terminal events. Success	Probability of surviving. Success (%)	Cumulative Success at the end of each interval	95% CI of cumulative success proportion	
							Lower limit	Upper limit
0	598	0	598	22	96	.96	0.9404	0.9796
5	576	**71**	540	17	97	.93	0.9104	0.9496
10	488	184	396	18	95	.89	0.8704	0.9096
15	286	93	239	10	96	.85	0.8108	0.8892
20	183	91	137	3	98	.83	0.7908	0.8692
25	89	38	70	2	97	.81	0.7512	0.8688
30	49	34	32	0	100	.81	0.7512	0.8688
35	15	15	7	0	100	.81	0.7512	0.8688

## Discussion and conclusion

This sample, including 312 patients and 598 ETT, demonstrated high tooth survival rates, especially during the first 20 years after treatment. In fact, at 10 and 20 years post treatment, the probability of survival for an ETT was 97% and 81%, respectively. These results are consistent with other clinical studies. After 10 years, Fonzar et al. [8] reported 93% survival rate, whereas Pirani et al. [15] and Fernandez et al. [16] reported 87.1% and 91.6%, respectively. After 25 years, the results from the present study indicated that 77% of ETT were still in painless function. These results were consistent with what was reported by Prati et al. [9] with a cumulative survival rate of 79% at 20 years but well beyond the 46% presented by Lee et al. [17] after 25 years. After 30 years of follow-up, the reported survival rate in this study was 76%. These results are in line with data from another study published by Mareschi et al. [18] which demonstrated 70% cumulative survival rates after 29 years. In this latter study, patients were treated by only one expert operator in a private practice with similar treatment procedures, which justifies the very similar findings. Regarding the reasons for tooth extraction, most teeth were extracted due to vertical root fracture (33.3% of the extracted teeth sample) and progression of periodontitis (40.2% of the extracted sample) (Table 6). These data are consistent with percentages reported by Mareschi et al. [18] and Fonzar et al. [8] who demonstrated that vertical root fracture and periodontitis were the most represented reasons for tooth extraction, respectively. It is noteworthy to highlight that no tooth was extracted due to endodontic inflammation.

**Table 6:** Distribution of the sample in relation to the causes of extraction and tooth type: 1, maxillary incisor; 2, mandibular incisor; 3, maxillary canine; 4, mandibular canine; 5, maxillary 1st premolar; 6, maxillary 2nd premolar; 7, mandibular 1st premolar; 8, mandibular 2nd premolar; 9, maxillary 1st molar; 10, maxillary 2nd molar; 11, mandibular 1st molar; 12, mandibular 2nd molar

Tooth type	n	Mx. Inc. 1	Mb. Inc. 2	Mx Can. 3	Mb Can. 4	Mx 1Prem. 5	Mx 2Prem. 6	Mb 1Prem. 7	Mb 2Prem. 8	Mx 1Mol. 9	Mx 2Mol. 10	Mb 1Mol. 11	Mb 2Mol. 12
Causes of extraction													
VRF	29	0	0	2	1	2	7	2	5	0	3	6	1
Periodontal disease progression	35	5	6	3	3	1	3	0	0	5	7	2	0
Caries	10	3	0	2	0	0	0	0	0	3	0	1	1
Crown fracture	13	1	0	0	0	1	0	0	1	2	1	5	2
Endodontic inflammation	0	0	0	0	0	0	0	0	0	0	0	0	0

Even though survival analysis is important to evaluate the performance of any therapeutic intervention, it is even more relevant to study the prognostic factors associated with this survival since these may guide treatment decisions and preventive measures. In this study, the logistic regression model identified PPD ≤ 5 mm as a protective factor (OR = 0.68), influencing the tooth survival distribution, as shown in Fig. 5c. In fact, at 30 years, only approximately 30% of > 5 mm PPD teeth were still in function, as compared to 80% of teeth with PPD ≤ 5 mm (Fig. 5). This finding is consistent with data from RCTs in which the odds of failure of ETT increased significantly in teeth diagnosed with mild (OR = 1.9) or moderate (OR = 3.1) periodontitis as compared to periodontally healthy teeth [19]. Similarly, it is well known that the presence of residual deep PPD (≥ 6 mm) was identified as the main risk factor for tooth loss after periodontal treatment [20, 21]. Another significant factor associated with tooth loss was pre-operative radiographic radiolucency. Teeth with radiographic radiolucency are almost two times (OR 1.87) as likely to be extracted than those without (Fig. 5b). This finding is in line with data from a very similar long-term study in which the presence of periapical radiolucency was correlated to higher odds (OR 1.9) of tooth extraction [18].

In contrast with other studies reporting molar teeth as a prognostic factor for tooth loss [1], tooth type was not significant in the present study. A reason for this difference may be related to the operating skills of the practitioner performing the endodontic therapy. There is evidence that suggests when it is carried out by an endodontist, there is a higher probability of tooth survival at 5 years, compared to a general practitioner (98.1% vs. 89.7%, respectively) [22]. In this investigation, all endodontic treatments were always performed by the same endodontist (GFV), which may also explain the lack of influence of the tooth type on survival rates.

The post-operative restorative factors that influenced tooth survival were the use of cast metal or fibre posts and the use of a night guard. Teeth with a cast metal post had two times (OR 2.13; CI 95% (1.137–4.015)) higher chance of being extracted. Conversely, the use of fibre posts was associated with 0.026 times lower chances of being extracted (OR 0.469; CI 95% (0.242–0.912)). These findings agree with other studies demonstrating a correlation between retentive metal intra-canal systems and lower survival rates [16, 23]. Similarly, the protective effect of fibre posts has been reported [24, 25].

While the protective role of a crown restoration after root canal treatment has been reported in some studies [23, 26] and systematic reviews [1], others have reported that placing a crown restoration on a ETT increased the risk of any complication by five times [27]. In the present study, crowned ETT did not present higher tooth survival. A possible explanation to this discrepancy may be related to the number of residual remaining walls, rather than the type of restoration [25]. Indeed, in this study, the selection of restorative therapy was based on the residual tooth structure, always selecting crown restorations when cusp coverage was needed. Furthermore, recent data based on systematic review and meta-analysis [28] evaluating the clinical performance of direct composite resin versus indirect restorations on ETT demonstrated no differences in tooth survival, in line with results from the present study. The importance of occlusal protection was also demonstrated in this study by identifying, in the logistic regression model, the use of a night guard as a protective factor influencing the tooth survival distribution (Fig. 5a).

In the present study, the overall success rates of ETT of 87.8% and 80.8% at the tooth and patient levels, respectively, were high and in line with data reported in the literature [8]. When analysing the cumulative endodontic success rates, reported values in the life table analysis were 93%, 85%, 81% and 81% after 10, 20, 30 and 37 years, respectively. These results are higher than what has been presented in systematic reviews with reported success rates of 82.8 % at 5 years [29] or in individual studies with success rates of 61% at 8 years [26]. Similarly, when looking at long-term follow-up, Lee et al. [17] observed 29% success rates after 25 years. These latter success rates are lower than the ones observed in the present study, which were 83% and 81% after 25 and 37 years, respectively. Several reasons may explain the major differences among the two studies, such as (I) biases in the method of visualisation of the lesion related to the two-dimensional radiographs, (ii) diverse criteria used to evaluate endodontic success, (iii) private practice-based versus teaching hospital-based study and (iv) different endodontic and restorative procedures [30, 31].

Like any retrospective observational case series, this study presents several limitations, largely dependent on the availability and accuracy of the data records. Despite this, an effort was made to include all patients attending the practice recall programme at least once per year with existing records, including more than 30 clinical and radiographic variables. Another important factor that must be considered is that due to the 5-year follow-up inclusion criteria, ETT with early (1–4 years) failures may have been lost during case selection. This selection bias, inherent to the retrospective nature of this evaluation, is a strong limitation that may justify the lack of extractions due to endodontic inflammation and consequently mask, in part, the overall data of survival of this case series. Notwithstanding this fact, the evaluation of two private practice-based studies using a protocol similar to the one utilized in the present investigation reported that the early failures in the 0–5 years’ time frame was approximately 2–3% of the entire sample (8, 18). These data should be clearly considered when drawing conclusions. Another aspect that needs to be considered is the fact that treatment protocols for restorative procedures and materials have changed slightly throughout the entire study period, and this is a potential confounding factor that may in part have affected the outcomes. It is also important to highlight that the external validity of this study is limited since a single operator in one clinical centre performed all the RCTs, and hence, the reported outcomes need to be extrapolated with caution to all clinical scenarios.

To the best of the authors’ knowledge, this is the longest follow-up study that demonstrates very high cumulative survival (68%) and success (81%) rates of ETT after 37 years of follow-up. These data, within the context of modern clinical dentistry, with a clear tendency to oversimplifying treatment plans and recurring to extraction of pathologically affected dentitions and replacing them with dental implants, should clearly encourage clinicians towards a conservative approach when treating the disease and maintaining the natural dentition.

In conclusion, within the limits of the present study, high long-term survival and success rates after primary RCT may be expected. The presence of deep periodontal pockets (≥ 6 mm), the lack of occlusal protection (no use of a night guard) and pre-operative apical radiolucency are the most significant prognostic factors associated with tooth extraction.

## Data Availability

The data that support the findings of this study are available from the corresponding author upon reasonable request.
